# Mechanical and Physical Regulation of Fibroblast–Myofibroblast Transition: From Cellular Mechanoresponse to Tissue Pathology

**DOI:** 10.3389/fbioe.2020.609653

**Published:** 2020-12-22

**Authors:** Mirko D'Urso, Nicholas A. Kurniawan

**Affiliations:** ^1^Department of Biomedical Engineering, Eindhoven University of Technology, Eindhoven, Netherlands; ^2^Institute for Complex Molecular Systems, Eindhoven University of Technology, Eindhoven, Netherlands

**Keywords:** fibroblast, myofibroblast, fibroblast-myofibroblast transition, mechanoresponse, fibrosis, homeostasis

## Abstract

Fibroblasts are cells present throughout the human body that are primarily responsible for the production and maintenance of the extracellular matrix (ECM) within the tissues. They have the capability to modify the mechanical properties of the ECM within the tissue and transition into myofibroblasts, a cell type that is associated with the development of fibrotic tissue through an acute increase of cell density and protein deposition. This transition from fibroblast to myofibroblast—a well-known cellular hallmark of the pathological state of tissues—and the environmental stimuli that can induce this transition have received a lot of attention, for example in the contexts of asthma and cardiac fibrosis. Recent efforts in understanding how cells sense their physical environment at the micro- and nano-scales have ushered in a new appreciation that the substrates on which the cells adhere provide not only passive influence, but also active stimulus that can affect fibroblast activation. These studies suggest that mechanical interactions at the cell–substrate interface play a key role in regulating this phenotype transition by changing the mechanical and morphological properties of the cells. Here, we briefly summarize the reported chemical and physical cues regulating fibroblast phenotype. We then argue that a better understanding of how cells mechanically interact with the substrate (mechanosensing) and how this influences cell behaviors (mechanotransduction) using well-defined platforms that decouple the physical stimuli from the chemical ones can provide a powerful tool to control the balance between physiological tissue regeneration and pathological fibrotic response.

## Introduction

**Figure 1:**
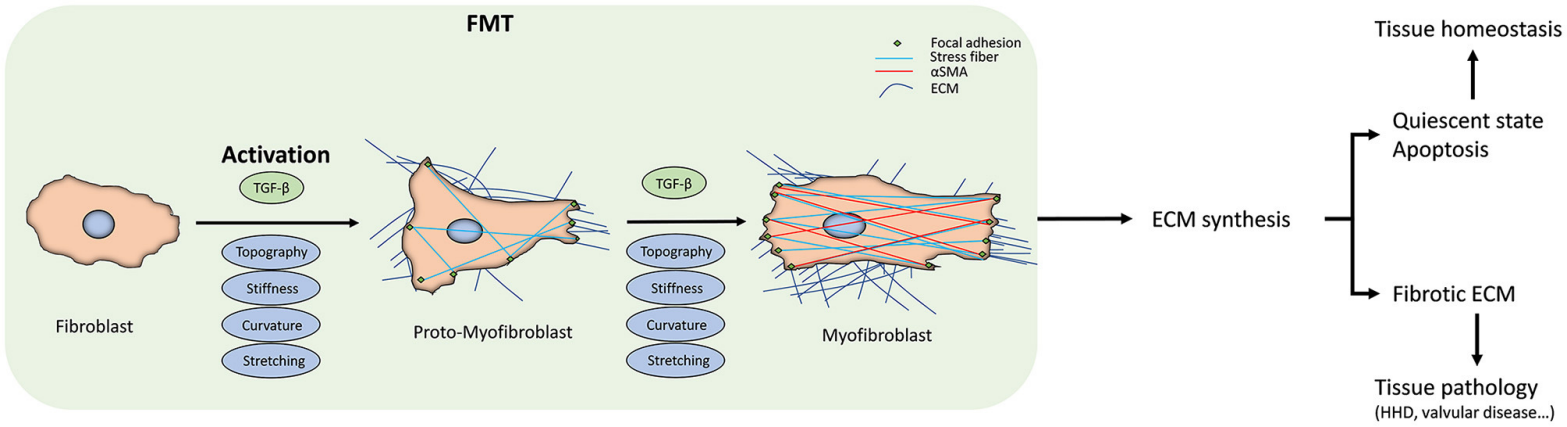
Fibroblast-to-myofibroblast transition (FMT). The scheme summarizes the FMT process, the corresponding changes in fibroblast behavior, and the downstream effects at the tissue level. The transition start from the fibroblast activation due to the different kinds of stimuli. The activation can sometimes be reversed or can proceed to the apoptosis of the myofibroblasts. When they escape these routes, due either to the persistent stimuli or to intracellular misregulations, FMT will lead to changes in the extracellular matrix (ECM) deposition and its architecture, driving the tissue to a pathological state. At the cellular level, FMT results in appreciable in the intracellular stress fibers and α-SMA expression.

Fibroblasts are cells belonging to the mesenchyme that are capable of producing and modifying extracellular matrix (ECM) components such as fibronectin and collagen (Kanekar et al., 1998). They are present in various tissues. For example, in neonatal and adult heart tissues, fibroblasts arise from endogenous cell populations via epithelial to mesenchymal transition (EMT) and from bone marrow derived cells (Visconti et al., 2006). Cardiac fibroblasts play a crucial role during fetal development and neonatal growth by contributing ECM to several specific structures of the heart (Figure 1) (Manso et al., 2009; Souders et al., 2009). In general, fibroblasts are flat and spindle shaped and can be easily distinguished from other cell types residing in the tissues, as fibroblasts lack tissue-specific functional hallmarks. Returning to the example of the heart tissue, the cardiac fibroblasts lack the basement membrane typical of the other cardiac resident cells (Kanekar et al., 1998).

Despite this, fibroblasts perform various critical functions in tissues and organs, such as generating ECM, actively migrating, and producing or degrading growth factors and cytokines that are fundamental for inflammatory cell response. Fibroblasts are also key players in several tissue-specific functions, such as ensuring normal heartbeat, where they form and maintain networks of junctions with several cell types, without which the tissue enters a pathological state (Camelliti et al., 2004, 2005; Baudino et al., 2006). As such, understanding their behavior within the tissue is a matter of high relevance, especially given that fibroblasts are a very common cell type throughout the human body. Fibroblasts have been extensively studied *in vitro* over several decades, partly because they can be easily derived from different tissues and aided by the simplicity of their *in-vitro* culture. There is evidence that fibroblast have to be activated to proliferate and migrate during specific pathophysiological conditions such as wound healing and fibrosis, and thus play an important role for development and repair of tissues (Gabbiani, 1996; Hinz et al., 2007). How the activation, phenotype transition, and migration of fibroblast take place in the contexts of injury response, tissue regeneration, wound healing, and fibrosis remains a key outstanding question.

One of the first responses after a stress in the tissue, such as in acute injures, is physical changes at the cellular and tissue level, such as tissue stiffening associated with changes in the ECM composition (Georges et al., 2007). These changes inevitably disrupt the mechanical homeostasis that underlies normal tissue architecture and function (Humphrey et al., 2014). Inflammatory signals such as transforming growth factor beta (TGF-β) and tumor necrosis factor alpha (TNF-α) are released after injury, which can lead to cytoskeletal remodeling that, in turn, alters cell-generated forces and cellular mechanical properties (Wang et al., 2001; Leung et al., 2007; Yang et al., 2011). When the injuries cannot be resolved and repaired, the response switches from wound healing to fibrosis.

Fibroblast-mediated fibrosis can affect every tissue of the body and is a frequent pathological feature of chronic inflammatory diseases. During this pathological process, homeostasis is disrupted and a variety of biochemical factors are released by inflammatory cells, which trigger fibroblasts to undergo a phenotypical change to become myofibroblasts, which in turn leads to a notable change in the tissue microenvironment. One critical pathway is the TGF-β pathway (Wynn and Ramalingam, 2012; Rockey et al., 2015). This pathway can strongly impact the transition of fibroblasts to a myofibroblast phenotype, which involves alpha smooth muscle actin (α-SMA) production with stress-fiber-like appearance, further leading to migration, proliferation, and production of ECM components such as collagen type 1 that changes the mechanical and physical properties of the environment. It was shown that increasing matrix stiffness, a phenomenon observed in aging tissue, leads to myofibroblast activation (Wang et al., 2006; van Putten et al., 2016). During dermal wound healing, the stresses within the tissues are reduced especially inside the wound bed, causing myofibroblasts to enter a quiescent state or initiate the apoptosis pathway (Desmoulière et al., 1997; Hinz et al., 2001b). On the other hand, splitting the wound or exposing the tissue to chronic mechanical stress keeps the myofibroblasts activated, leading to the opposite response, i.e., preventing healing and promoting scar formation (Aarabi et al., 2007; Gurtner et al., 2011). Similarly, during wound healing, myofibroblast can be either inactivated, going toward a more quiescent state, or continue with its normal functioning, leading the tissue in which it resides along the fibrotic pathway. This risk associated with myofibroblasts escaping inactivation and overcoming the apoptosis control is always present; an example of controlled escaping is in the CCl_4_ rodent liver tissue model, where hepatic stellate cells can turn into myofibroblasts (Kisseleva et al., 2012).

As we shall discuss, the evidence indicates a complex, dynamic interplay between fibroblasts and the extracellular matrix in the tissue, where cells alter the properties of the environment and, at the same time, changes in the substrate mechanical and physical cues lead to changes in cellular organization and behavior (Jaalouk and Lammerding, 2009; Kurniawan et al., 2016). Sensing of physical extracellular cues and the subsequent dynamics of the interaction between cells and the ECM regulate the downstream mechanotransductive events, causing a variety of nano- and micro-topography-sensitive cellular behaviors, including cell adhesion, morphology, proliferation, gene expression, self-renewal, and differentiation (Lemischka and Moore, 2003; Kingham and Oreffo, 2013). In light of these, in this review we highlight how a better knowledge of how physical/mechanical stimuli can influence the phenotype transition of fibroblasts can provide us with a better control on this process and allow us to revert in more efficient way the fibrotic tissue response, thereby presenting an important step forward to treat fibrotic pathologies.

## Fibroblast-to-Myofibroblast Transition

A key step in wound healing, but also in fibrotic pathological diseases, is the activation of the fibroblast to become myofibroblast, where they escape the entrance to a quiescent state or the apoptosis pathway (Gabbiani et al., 1971). This phenotype transition is defined as Fibroblast-to-Myofibroblast Transition (FMT). The influence of FMT has received significant attention in the context of diseases such as bronchial asthma (Michalik et al., 2018). Some tissue-specific FMT events have been identified, such as increased collagen deposition within the subepithelial basement membrane in asthma, although these events do not fully explain the variations in the severity of asthma (Chu et al., 1998). Here, therefore, we will focus on the shared features of FMT and factors that promote FMT, drawing examples from different tissues and tissue pathologies.

**Figure 2:**
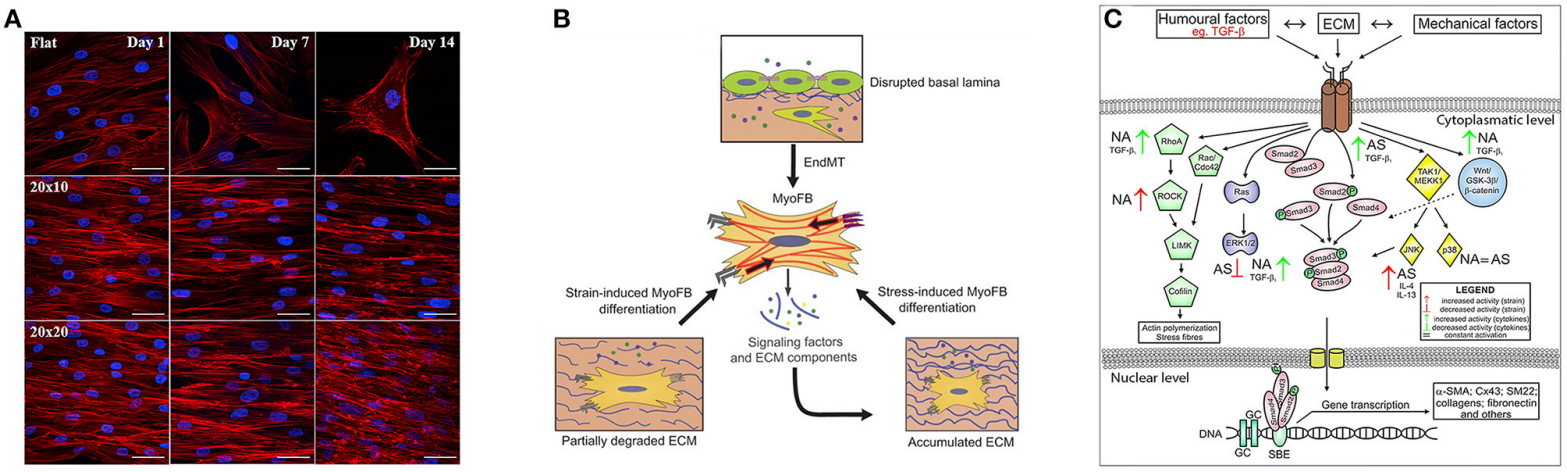
Key signatures of FMT. **(A)** Influence on cytoskeletal arrangement and cardiomyogenic differentiation of the substrate that presents pattern at different time points. Along the first row the cells reside on flat surface while the following two present same kind of grooves but with different dimensions. Image was adapted with permission from Gu et al. (2017). **(B)** Schematic illustration of the mechanostimulation that lead to myofibroblast differentiation. The upper part shows that endothelial cells lose their endothelial markers. The lower part shows that mechanical factors, such as the degradation or production of ECM that alter tissue stiffening, can induce the differentiation, for example in cardiac fibroblasts. Image was adapted with permission from Schroer and Merryman (2015). **(C)** Different fibroblast activation pathways through biochemical and mechanical factors in asthmatic (AS) and not asthmatic (NA) patients. Image was adapted with permission from Michalik et al. (2018).

Broadly, FMT can be subdivided into 2 stages. The fibroblast first become activated to a proto-myofibroblast phenotype, followed by a second stage completing the cell phenotype transition (Tomasek et al., 2002; Hinz and Gabbiani, 2003). During the initial transition stage, distinguishing the normal fibroblasts from proto-myofibroblasts is very difficult, but if the elevated mechanical, physical, and biochemical stresses due to the injuries continue to be present in the tissue, they start the polymerization of α-SMA-containing stress fibers (Figures 2A,B). The hallmarks of the full transition into myofibroblast are the expression of β-cadherins, the formation of mature focal adhesions (FAs), and reduction in migration and proliferation, with increased contractility (Hinz and Gabbiani, 2003; Hinz et al., 2004; Ward et al., 2008). The mechanical tension of the wound and the presence of growth factors further push toward the phenotype transition from fibroblast to myofibroblast (Balza et al., 1988). Interestingly, the formation of stress fibers that promote cell motility can also be induced by the presence of growth factors (Malmström et al., 2003), suggesting the role of environmental humoral stimuli in FMT.

### Environmental Stimuli Affecting FMT

Humoral stimuli (Figure 2C) have been generally believed to be fundamental to drive the phenotype transition of fibroblast, especially the role of TGF-β growth factor. TGF-β is present in 3 different isoforms (TGF-β_1_, TGF-β_2_, and TGF-β_3_) that are secreted by cells into the extracellular space (Minshall et al., 1997; Batra et al., 2004; Howell and McAnulty, 2006; Rahimi and Leof, 2007). TGF-β may have pro- or anti-apoptotic effects on epithelial cells (Al-Alawi et al., 2014) and can induce EMT in airway epithelial cells from asthmatic subjects (Hackett et al., 2009). It is well-documented that TGF-β can trigger FMT in asthmatic subjects (Sagara et al., 2002; Le et al., 2007; Luo et al., 2014) and in *in-vitro* cultures (Minshall et al., 1997; Batra et al., 2004; Howell and McAnulty, 2006; Boero et al., 2007; Milara et al., 2012). It was furthermore shown that other growth factors have a well-coordinated activity with TGF-β to promote FMT, such as CTGF (also known as CCN2) (Kular et al., 2011), PDGF, which increases the number of migrating cells and encourages phenotypical shifts of lung fibroblast toward myofibroblasts (Malmström et al., 2003), as well as NGF (Bonini et al., 1996) and IGF-1 (Yamashita et al., 2005; Boero et al., 2007) in different tissues.

Another kind of FMT-inducing stimuli is related to an elevated influx of immune cells associated with increased vascular permeability, and the subsequent release of cytokines and chemokines. Especially the role of interleukins during inflammatory response and how they are correlated to FMT is well-understood. For example, when stimulated by IL-4 and IL-3, the expressions of α-SMA in human lung fibroblasts are increased depending on the interleukins concentration and in a time-dependent manner (Hashimoto et al., 2001; Saito et al., 2003).

More recently, mechanical stimuli have also been demonstrated to have a key role in FMT (Tomasek et al., 2002; Balestrini et al., 2012; Hinz et al., 2012; Darby et al., 2016). Several *in-vitro* studies have shown that mechanical stress within the cellular environment, induced for instance by different mechanical and physical properties of collagen gels, is one of the factors that controls the shift in the fibroblast phenotype and cell fate (Arora et al., 1999; Hinz et al., 2001b; Wang et al., 2003; Choe et al., 2006; Balestrini et al., 2012). However, at the moment, there are still very few results on the direct impact of mechanical factors and their influence in FMT. There is evidence that mechanical stress leads to an increase of the ECM proteins and proteoglycan content (Breen, 2000; Ludwig et al., 2004; Le Bellego et al., 2009; Manuyakorn, 2014; Manuyakorn et al., 2016) and in recent studies it was demonstrated that mechanical properties of the microenvironment in which the cells reside, such as lung, bronchial, neuronal and cardiac tissues, have an active role on cell fate and their development (Tschumperlin, 2013; Michalik et al., 2018; Park et al., 2018). These studies suggest that biochemical, mechanical and physical factors in the microenvironment take part in a regulatory network that leads to different cell fate by specifically inducing intracellular changes also at a mechanical level, inducing the completion of the phenotypic transition from proto-myofibroblast to myofibroblast.

### Role of Physical Stimuli in FTM

#### Cell Phenotype Is Driven by Physical and Mechanical Properties of the Environment

*In vivo*, cells are embedded in a complex ECM during both development and normal homeostatic maintenance, where ECM fibers present chemically and structurally intricate contact interfaces. Within this intricate ECM network, fibroblasts and other cell types drive ECM remodeling by deforming, reorienting, and degrading the ECM fibers, as well as depositing new ECM (Zamir et al., 2000; Shieh et al., 2011). These events are critical for tissue morphogenesis and maintenance. To study these contact interfaces systematically, minimal *in-vitro* model systems using either microfabricated substrates, controlled deposition of ECM fibers, or structured protein patterns have been developed (Kurniawan and Bouten, 2018).

Experiments performed using adipose stromal cells (ASCs) cultured in collagen matrices of different architectures show that matrices with thicker fibers promote ASC phenotype transition into myofibroblast through regulation of VEGF and IL-8 secretion (Seo et al., 2020). Intriguingly, nanoscale changes in the ligand spacing of model ECM fibers were shown to influence the collective cell behavior and overall characteristics, such as action-potential propagation in cardiac myocytes, on the scale of centimeters, suggesting effects on the ECM organization over six orders of magnitude of length scale (Kim et al., 2010). Recent works from our group using patterning of ECM proteins have furthermore shown that various morphological features of myofibroblasts that are relevant for FMT, such as cell area, shape, elongation, and alignment, are sensitively governed by the ECM patterns in a length-scale-dependent manner (Buskermolen et al., 2019, 2020). Moreover, the ECM architecture at the microscale induces different cellular events that activate a mechanical feedback loop whereby cell-generated forces lead to matrix remodeling, which in turn induces mechanotransductive processes and thus influencing the cell-generated forces again, by modulating the cell's capability to form and mature FAs as a result of changes in the stiffness of the substrate (Hall et al., 2016; Sapudom et al., 2019). Taken together, these findings clearly show that physical cues from the environment can strongly influence the phenotype of tissue-resident fibroblasts, which in turn can shape tissue homeostasis.

Another way that microenvironmental cues can affect tissue homeostasis is through changes in cell composition due to cellular movements. A relatively well-recognized consequence of the abovementioned mechanical feedback loop is the family of cellular “taxis” responses triggered by the ability of cells to sense chemical, mechanical, electrical stimuli gradients in the environment. These taxis responses include chemotaxis (sensing to spatial gradients of chemical factors) (Devreotes and Janetopoulos, 2003), haptotaxis (sensing of the surface-bound ECM proteins densities) (McCarthy and Furcht, 1984; Isenberg et al., 2009), durotaxis (sensing of substratum rigidity) (Lo et al., 2000), galvanotaxis (sensing of electric fields) (Mycielska and Djamgoz, 2004), and curvotaxis (sensing of cell-scale curvature variations) (Pieuchot et al., 2018). These sensing machineries can be locally activated within the tissue microenvironment, triggering specific mechanotransductive pathways that not only can instigate FMT directly, but also can promote active migration of fibroblasts and myofibroblasts into and out of the tissue. Using artificial engineered substrates that mimic the chemical, mechanical, and physical properties of highly organized ECM fibers, and so controlling their spatial density, it was shown in a recent study that the fiber density variation can be sensed by fibroblasts, and interestingly different cell types exhibit different sensitivities along a density gradient depending on their cortical stiffness (Kim et al., 2012). Interestingly, skin fibroblasts have bidirectional guidance from the highest and the lowest density areas toward an optimal one. This suggests that a topotactical guidance depending on ECM density is present. Indeed, cells tend to move toward the direction that allows them to make the largest contact area with the substrate (Park et al., 2018). Of note, fibroblast sensitivity to the taxis guidance cues can vary with its activation state; indeed, myofibroblast migrate differently depending on the mechanical and physical properties of the ECM (Berk et al., 2007). During wound healing, fibroblasts are directed chemotactically by the presence of TGF-β_1_ in the microenvironment where the homeostasis is disrupted (Chen et al., 2020) and can afterward shift their phenotype to myofibroblasts, leading further to cell migration toward the wound site, thereby increasing the local myofibroblast subcellular population (van Caam et al., 2018).

Through these cellular response to the physical cues in the environment, pathological features can emerge and progress. A high density of myofibroblasts and a different ratio between ECM components can be found in the bronchial and transbronchial biopsies of advanced asthma patients, compared to those of the patients with controlled and treated asthma symptoms (Weitoft et al., 2014). These are caused by activation of myofibroblasts within the tissue, which start a positive loop to retain their activated state instead of entering a quiescent state, as well as the associated regulation of metalloproteases MMPs and their regulators tissue inhibitors (TIMPs) secretion, thereby allowing the progression of the pathological state.

Interestingly, specific mechanical requirements have been found using a 2D *in-vitro* platform that the substrate must satisfy in order to initiate FMT. In particular, the substrates have to present a Young's modulus of at least 3 kPa, which allows the cells to produce large, mature integrin clusters that enable the full phenotype transition (Balestrini et al., 2012). Moreover, depending on the cell type studied, it can happen that stiffer culture substrates with a Young's modulus higher than 20 kPa are needed to continue the mechanotransductive machinery required to drive the phenotype transition of fibroblasts. Similarly, during *in vitro* wound healing assays, FMT requires a stiffness threshold in range of 25–50 kPa (Balestrini et al., 2012). These studies further emphasize the importance of physical and mechanical interactions with the ECM during FMT.

#### The Contact Events Start the Signal Transduction

Contact events with ECM and maturation of adhesion complexes are the first key steps in cell–ECM interactions that allow the regulation of cell functions such as growth, differentiation, and disease (Hynes, 2002; Geiger et al., 2009). The adhesion complexes arise as nascent adhesions (Alexandrova et al., 2008; Choi et al., 2008) that reach the dimensions of ~110 nm (Bachir et al., 2014; Changede et al., 2015; Changede and Sheetz, 2017). Their maturation is then promoted through outside-in mechanotransduction mechanism from the matrix (Wolfenson et al., 2016; Saxena et al., 2017a,b).

When cell membrane receptors bind a ligand in the ECM substrate, the intracellular tail of the B subunits of integrins binds talin, a mechanoprotein in closed conformation. Talin links the B subunits with F-actin and, due to the force exerted by myosin II through F-actin, switches to an open conformation, consequently exposing binding site for vinculin, another protein that confers stability to this complex, the so-called “molecular clutch” (Sheetz, 1974; Geiger et al., 2001; Swaminathan and Waterman, 2016). First, vinculin binds to the binding site along talin, which is in open confomation, nearby the link between integrin β-tail and talin. Subsequently, vinculin binds in the same way along talin, but in proximity of the link between talin and F-actin. If this clutch can support the loading force exerted by the myosin II on the cell membrane receptors, the maturation of FAs lead to mechanotransduction depending on properties of the environment where the cell reside in Sheetz (1974), Geiger et al. (2001), and Swaminathan and Waterman (2016). The maturation of the FAs has been directly linked to ligand spacing (Dalby et al., 2014) as well as substrate stiffness (Oria et al., 2017), showing that depending on the Young's modulus of the substrate, the minimum ligand spacing necessary to lead at maturation of adhesion complexes can change. The molecular clutch mechanism can therefore instigate FMT through these mechanosensitive responses at the cell–substrate contact interface.

#### ECM Composition Regulates Fibroblast Mechanosensing

The ECM composition is also a hallmark of the pathological state of the tissues. Depending on the abundance of its components, the ECM can present different microarchitectures, leading to different mechanical and physical properties. Importantly, interactions between the cells and the different ECM components can directly regulate cell behavior such as migration and development (Park et al., 2018; Changede et al., 2019; Nastały et al., 2020).

One of the main components of the ECM that has been shown to play an important role in promoting FMT is fibronectin splice variant ectodomain A (ED-A-FN), which is upregulated in pulmonary disorders such as asthma (Larsen et al., 2006; Ge et al., 2015). Fibroblast from lung ovalbumin treated mice that lack the ED-A-FN present a reduced tendency to proliferate and migrate, and very interestingly display a lower α-SMA expression as well as less collagen deposition with impaired TGF-β_1_ and IL-13 release, which are all hallmarks of a phenotype transition (Kohan et al., 2011). This suggests that the composition of the ECM can influence the mechanical stress in the tissue and thus affecting cellular phenotype transition. Another evidence is the role of the fibulin-1, a glycoprotein related to the stabilization of other protein group in ECM that is also known to be a marker for bronchial asthma (Lau et al., 2010; Giziry et al., 2017), indicating that the enhanced stability of ECM increases the propensity of fibroblasts to FMT.

It was demonstrated that enhancing actomyosin-mediated cell contractility can induce stromal cell mechanoactivation, leading to adipose stromal cells turn into myofibroblasts (Seo et al., 2015). This transition, in turn, leads to changes in the cellular environment through deposition of more fibronectin as well as deformation of the fibronectin network, partially unfolding the fibronectin molecules (Wan et al., 2013; Wang et al., 2017).

#### Mechanotransduction Leads to Distinct Internal Cellular Rearrangements

Following the mechanosensing events described above, the mechanical signals are transduced to elicit a variety of cellular responses that are also reflected in the alterations of internal cell organizations relevant for the progression of FMT. Here we highlight a few notable findings that exemplify this concept.

It has been recently demonstrated that cell behaviors that are implicated in FMT, such as migration, can be influenced by the curvature of the substrate (Pieuchot et al., 2018; Werner et al., 2019). This is especially interesting as curvature is a common geometrical feature of *in-vivo* tissues and organs (Callens et al., 2020; Werner et al., 2020). Convex spherical surfaces have been shown to cause a compression of the cytoskeleton on the nucleus, increasing the contact area between cell and substrate (Werner et al., 2017). Thus, cell nuclei were flattened and stretched over the convex surface, even resulting in a bean-like nuclear morphology. This effect on nuclear morphology can further translate to changes at transcription level. In a recent study, it was observed that, depending on the adhesion area, fibroblasts alters their cytoskeletal tension to the nuclear envelope; small substrates areas leads to an increased histone acetylation levels with a decreased nuclear volume (Alisafaei et al., 2019). Moreover, the motility of cells is regulated by the organization of stress fibers (SFs), but in curved environment the fibroblasts present a different SF organization with respect to those on planar substrates. A negative curvature polarize the cells and direct cell migration (Bade et al., 2018). Therefore, rearrangement of the cytoskeleton through mechanotransductive machinery leads to changes in the nucleus polarity and positioning within the cell, influencing cell migration (Vassaux et al., 2019; Moure and Gomez, 2020). Indeed, both F-actin and focal adhesion distributions were strongly influenced by this repositioning of nuclear compartment (Nastały et al., 2020). These findings provide intriguing insights that physical reorganization of the intracellular structural components and mechanotransductive players, which can be induced by changes in the tissue morphology, can directly affect FMT.

Further evidence of the importance of the mechanosensing in governing intracellular organizations and cellular response can be observed by direct perturbations to the mechanosensing apparatus. When ASCs that are undergoing FMT are treated with Y27632, which inhibits ROCK and reduces α-SMA levels (Seo et al., 2015), as well as diminishing the capability of the cells to sense the environment by inhibiting the receptors to TGF-β, the cells exhibit a decrease in myofibroblast transition and moreover reduced VEGF and IL-8 secretion. On the contrary, treating these cells with blebbistatin influences their morphology, confining the adhesions to the extreme cell periphery, causing actin stress fiber formation and enhancing contractility, thereby stimulating ASC myofibroblast transition (Seo et al., 2020). Consistent with this link between cell mechanosensing, force generation, mechanical properties, and organization, it is also increasingly recognized that ECM viscoelasticity, non-linear elasticity, and fiber rearrangement play a central role for cell behavior such as proliferation and multilineage differentiation (Baker et al., 2015; Chaudhuri et al., 2016; Das et al., 2016; Xie et al., 2017; Matera et al., 2019; Vining et al., 2019). Tuning the structural and mechanical properties of hydrogels has been shown to lead to different types of cellular organization and responses (Goh and Holmes, 2017; Herum et al., 2017).

With regard to active mechanical cues such as stretching, in recent study it was shown that exerting stretching on myofibroblasts lead to the maintenance of the shifted phenotype through the activation of the release of endogenous latent TGF-β_1_ (Walker et al., 2020). On the other hand, when treated to block the release of TGF-β_1_, stretched cells maintain their phenotype, exhibiting comparable contractility and stiffness as in static cultures. This suggest that cyclic stretching can be responsible to maintain the myofibroblastic phenotype, leading to chronic fibrosis (Walker et al., 2020). Furthermore, under stretch, TGF-β_1_-treated cells showed further alignment to static conditions, as well as increased gel compaction (Walker et al., 2020). This demonstrates the capability of mechanical stretch of the substrate to change the sensitivity of the cells to biochemical stimuli present within the environment. The stretching causes a downregulation of the ECM proteases, leading to an increase of collagen-I associated peptides secretion. This is consistent with the concomitant increases of inflammatory and fibrotic response of the tissue (Sun et al., 2016; Rogers et al., 2020).

Taken together, these studies highlight the importance of better characterization and quantification of the involvement of mechanical and physical properties of the environment in which the cells reside, giving attentions not only on the mechanotransductive machinery involved, but also achieving a better control on this machinery through the passive function of the substrates and the active adaptation of the cells.

## Dissecting the Cell–Substrate Interaction Events in FMT

The previous sections have unequivocally established the importance of changes at the mechanical level of the cell in FMT, from the first contact event with the substrate to the influence on cellular behavior. During this phenotype transition, a variety of humoral and mechanical cues from the substrate drive the fibroblast to myofibroblast transition via a proto-myofibroblast state. When the transition completes, the myofibroblasts present α-SMA containing stress fibers and an enhanced contractility. To complete such transition, the mechanical and physical properties of the environment play an active role in inducing intracellular changes. These cellular and intracellular events are overall interconnected through the mechanotransductive machinery, starting from the mechanosensing exerted by the fibroblasts. Thus, the dynamic interplay between the ECM and the cell seems to play a central role in cytogenesis, especially during FMT.

Efforts to understand the cellular mechanosensing and mechanotransduction mechanisms have gained significant attention since it became known that these are involved in cell differentiation processes (Yim and Sheetz, 2012; Dalby et al., 2014; Iskratsch et al., 2014; Murphy et al., 2014). Importantly, differentiation and FMT share many regulatory pathways that direct the expression or the release of factors involved in cell metabolism or cell fate, such as α-SMA production and the release of TGF-β. In the initial contact events with the substrate, mechanosensing and mechanotransduction of the physical signals in the environment leads to the maturation of the FAs only if the environment properties (ligands spacing and stiffness) satisfy the requirements to drive the changes. In addition, cellular sensing of extracellular topographical cues through nanoscale architecture causes a multitude of nanotopography-sensitive cellular behaviors, including cell adhesion, morphology, proliferation, gene expression, self-renewal, and differentiation (Lemischka and Moore, 2003; Kingham and Oreffo, 2013). This is possible though integrin-mediated sensing of mechanical and physical features of the microenvironment (Geiger et al., 2009; Dalby et al., 2014; Chen et al., 2015; Humphries et al., 2015) and will lead to intracellular rearrangements of the cytoskeleton and alteration in the mechanical proprieties of the cell, a key step in FMT. At the same time, the cell contractility is enhanced, causing the cell to release latent TGF-β and pushing toward FMT. Thus, dynamic interplay between the cells and the ECM induces cytoskeletal rearrangements that will simultaneously cause changes in the force transmission, cytoskeletal organization, and mechanical properties of the cell and its nucleus, as well as of the ECM in tissues (Jaalouk and Lammerding, 2009; Kurniawan et al., 2016; Nastały et al., 2020).

The importance of considering the cell–substrate interaction events in FMT should also be considered in light of the fibroblast heterogeneity in different tissue microenvironments. Indeed, fibroblasts exhibit differing functional identities, including the composition and expression profile of the intracellular macromolecules, depending on the tissue where they reside (Lynch and Watt, 2018; LeBleu and Neilson, 2020). *In-vitro* culture entails loss of most of mechanical and physical stimuli normally present in the tissue-specific microenvironments (Lynch and Watt, 2018), which affect not only the mechanical properties of the cells, but also cellular functions such as polarization (Nastały et al., 2020). This suggests a possible involvement of mechanotransduction in the regulation of gene expressions. As will be addressed in section Coupling of Mechanical and Physical Cues of the Substrates, combinations of different local physical and mechanical stimuli that are sensed by cells in a physiological environment, such as roughness, topography, stiffness, and stretching, could influence the functional identities of the fibroblasts, further leading to heterogeneity in the cell population.

Taken together, studying the mechanical interactions between cells and the substrate at the cell and tissue levels is critical, not only to start recognizing how much the environment is involved in physiological processes, but also to better understand how environmental features can be manipulated to speed up or slowdown pathological processes. To do so, *in-vitro* biomimetic substrates have become an invaluable toolset as a way to simplify the complexity of the *in-vivo* cell–substrate interactions.

## Production of Substrates With Well-Defined Physical and Mechanical Characteristics

**Figure 3:**
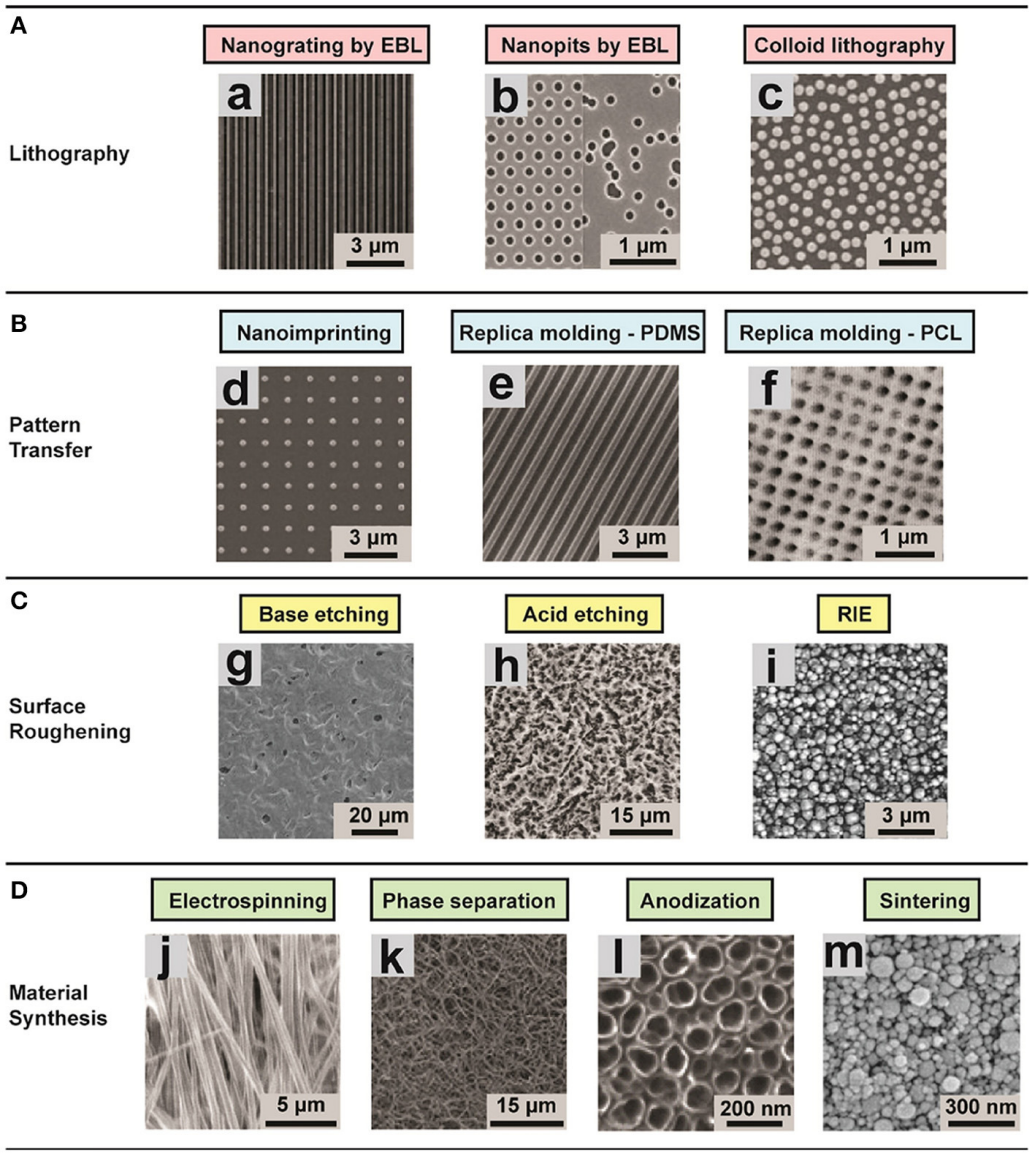
Representative images of different substrates (ordered and disordered) created using various techniques for studying the role of environmental effects on FMT. **(A)** Lithographic techniques: (a) nanogrooved silicon substrates with 70 nm wide ridge and 400 nm pitch; (b) arrays of 120 nm-diameter, 100 nm-deep nanopits on silicon substrates; (c) self-assembly of 110 nm-diameter nanoparticles. The substrates present a ordered shape. **(B)** Pattern transfer techniques: (d) Nanostructured polyurethane acrylate (PUA) surface with a patterned array of nanopillars fabricated; (e) PDMS nanograting; (f) PCL surface with nanopits with various scales. **(C)** Surface roughening, obtaining disordered topographies: (g) Nanostructured PCL with feature dimensions of 50–100 nm; (h) surface roughness R_a_ = 0.87 ± 0.03 μm; (i) surface roughness of 100 nm. **(D)** Disordered topographies: (j) Aligned nanofibrous hydroxybutyl chitosan (HBC) scaffolds; (k) Nanofibrous PLLA matrix with an average fiber diameter of 148 ± 21 nm and a porosity of 92.9%; (l) self-aligned TiO2 nanotubes with a diameter of 100 nm; (m) nanostructured alumina substrates with 24 nm grain-like structures produced with different physical and chemical synthesis. Image adapted with permission from Chen et al. (2015).

To better understand the role of physical and mechanical cues in the environment in FMT, a multitude of versatile substrate fabrication techniques have been developed and applied in the attempt to produce substrates that accurately mimic aspects of the ECM properties. In this section, we summarize commonly used methodologies for creating substrates with well-defined physical and mechanical features of ECM. Here we pay particular attention to the accessible length-scale target and characteristics (Figure 3), while referring readers who are interested in the detailed working principles and practical aspects to the original articles describing the individual methods. Moreover, we highlight the emerging efforts to use a combination of these cues to better mimic the physiological condition of the tissues.

### Mimicking ECM Topography

The aim of the approaches that focus on topography is to understand the impact of substrate micro- or nano-topography on mechanotransductive processes, and to exploit these substrates to control cell behavior. Various fabrication technologies have been adapted to the needs of cell biology (Dalby et al., 2014; Chen et al., 2015; Crowder et al., 2016; Chighizola et al., 2019). One of the most commonly applied group of techniques for structuring the surface is based on lithography. In this group, there are three main methodologies: photolithography, electron beam lithography (EBL), and colloidal lithography.

The optical lithography works by transferring the pattern that is required onto a photosensitive emulsion (photoresist) on a substrate. This approach can be very useful for creating adhesion patterns and controlling cells organization (Dalby et al., 2007; Bettinger et al., 2009). The main limitation with optical lithography is the requirement that the starting layer on which the features will be built must be a stiff flat surface, which precludes fabrication of 3D structures. Prefabricated structured rigid molds can be used in pattern transfer methods to print the mold features to other materials with high efficiency and fidelity (Guo, 2004; Pandey et al., 2019). The most common techniques are nanoimprinting and replica molding (Chen et al., 2015). These methodologies can be exploited to transfer patterns with resolution of a few hundred nanometers. In practice, the procedure for replica molding require baking time, while one of the bottlenecks of nanoimprinting is to control the demolding after the heating up to transfer the pattern. Normally these approaches are used in combination with microfluidic devices. Another methodology that does not necessitate templates is surface roughening that can be achieved, for example, by physical or chemical etching (Boyan et al., 1998; Thapa et al., 2003). Both methods can be applied to large surface areas, but an accurate control of the feature size is challenging (Chen et al., 2015). Surface roughening is a very useful technique that allows study of cell sensing of substrates with well-defined surface roughness.

In general, the mold must present precisely defined topographies that are transferred to the substrate. For example, to exploit the nanoimprinting lithography, the molds are produced using PDMS (Odom et al., 2002), polyurethane acrylate (Kim et al., 2003), and “hard-PDMS” (Schmid and Michel, 2000). In addition, there are some efforts directed toward the production of soft molds with an improved modulus and solvent resistance, although this has the drawback of reduced durability due to the temperature during the pattern transferring (Ro et al., 2011). Moreover, these kind of molds are produced starting from master inorganic templates, normally metals or ceramics (Albrecht et al., 2015). The bottleneck in their production is the complexity of the procedures.

The optical lithography approach can be applied at large scale and high throughput due to the very quick transfer of topography from the mold to the substrate. A modern lithography tool is able to produce till 300 mm/h patterned wafers with roughly 50 nm 2D pattern resolution, achieving a pixel throughput of 1.8T pixels/s. The achievable resolution of this method is determined by the UV light wavelength, as well as by the capability to reduce diffraction at the mask apertures by reduction lenses that capture higher order diffraction light. However, going beyond sub-100 nm resolution is very challenging (Chen et al., 2015).

In EBL, electrons are used instead of photons in order to improve the spatial resolution of the lithography, enabling a resolution of <10 nm (e.g., for periodical line patterns) (Michishita et al., 2014). This allows a very precise mimicry of nanotopographical ECM features (such as collagen fibers with lengths on the order of 10 μm; Buehler, 2006), but at the same time limiting the scale of surface area and throughput that can be fabricated with reasonable time/cost efforts (Chen et al., 2015). The above methodology is a top-down approach, meaning that the topographies are transferred to the substrate of interest through the usage of a mold. In practice, this approach is relatively time consuming. An alternative method is a bottom-up approach, which can produce ordered structures over large area in a cost-effective manner (Yamada et al., 2017). Another bottom-up approach is colloidal lithography, in which colloidal nanoparticle with crystal structures self-assemble on planar surfaces (Yang et al., 2006). These colloidal nanoparticles can then by reduced by etching. With this technique, one can achieve high throughput of nanometric features, but without an accurate control of the spatial pattern (Chen et al., 2015).

Furthermore, several material synthesis methods can be exploited for tissue engineering, such as electrospinning, phase separation, anodisation, and sintering, which have been described in detail in dedicated review articles (Zhang and Ma, 1999; Li and Xia, 2004; Park et al., 2007; Smith et al., 2008; Dulgar-Tulloch et al., 2009; Bhardwaj and Kundu, 2010).

### Coupling of Mechanical and Physical Cues of the Substrates

More recently, the importance of examining the effects of multiple mechanical and physical cues simultaneously presented to the cells has been increasingly recognized, as efforts are made to bridge the minimalistic model systems and complex *in vivo* situations. For example, in the study of Oria et al., the authors studied the ligands spacing coupled to the stiffness of the substrate, in this case a simple 2D protein patterning hydrogels allowing to control the Young modulus and the disposition of the ligands (Oria et al., 2017). The results indicate that finding the right combination can lead to the activation of mechanotransductive pathway, allowing the force loading on molecular clutches.

Fibroblasts have also been shown to sense the mechanical stiffness of the substrate. In fact, it is well-documented that, between the physiological and pathological states of the tissue, there is a significant difference in stiffness. This is caused by changes in the microenvironment composition, which in the pathological state is higher in collagen I and reduced in collagen III (Herum et al., 2017). Culturing fibroblasts on polyacrylamide gels with stiffness mimicking the pathological state of breast tissue (20 kPa) resulted in larger cell spreading area compared to on stiffness mimicking the physiological ECM (1 kPa) (Schwager et al., 2019). Moreover, α-SMA content increased on the stiffer substrate, suggesting that the fibroblast activation can be promoted by matrix stiffness (Schwager et al., 2019). When cardiac fibroblasts were cultured on hyaluronic acid gels with different stiffnesses ranging from the healthy myocardium (8 kPa) to the infarcted state (20–100 kPa), where the *in vivo* presence of myofibroblast is known to be higher, significantly reduced formation of α-SMA was observed on the softer substrates (corresponding to the healthy myocardium stiffness). In addition, the FAs on these substrates were small and peripheral, whereas on the stiffer substrates the FAs were bigger and distributed throughout the cell membrane (Herum et al., 2017). The mechanical properties of the ECM therefore seems to play an important role in the maintenance of the quiescent fibroblast phenotype and the FMT, highlighting the relevance of coming up with methods to fabricate substrates with tunable stiffness in the range relevant for physiological tissues.

Another kind of physiologically relevant mechanical stimulus that can be recapitulated *in vitro* is stretching. Tensile testing has been used to stretch silicon substrates on which cells have been allowed to adhere. This technique comprises an electronic control console and a loading frame with a load capacity of 2.5 N in tension or in compression (Boccafoschi et al., 2007). This stretching method can be combined with topographical cues to obtain different cell responses such as different intracellular rearrangements and adhesion patterns. For example, in the study by Gu et al., the authors analyzed the effects due to the simultaneous presence of protein patterns and cyclic stretching on the cardiomyogenic differentiation of hMSCs (Gu et al., 2017). In our group, we have examined the effect of stretch in combination with shear flow in a vascular construct that mimics the mechanical environment in cardiovascular tissues (van Haaften et al., 2018). This approach has revealed the distinct roles of stretch and shear in governing myofibroblast activity and neotissue production, both directly (van Haaften et al., 2018) and indirectly through crosstalk with immune cells (van Haaften et al., 2020; Wissing et al., 2020). Furthermore, a combination of substrate protein patterning, substrate stiffness, and mechanical stretching has been studied to push toward the complete FMT by stimulating the release of latent TGF-β (Walker et al., 2020).

This kind of coupling between mechanical and physical features of the environment seems to be the key to reach in a more controlled way different cell fates, by only exploiting the physical and mechanical characteristics of the substrates.

### 3D Environments for Studying Fibroblast Activation

Mimicking of physiological environment to better understand cellular responses and gain fundamental insights to prevent pathological outcome is getting increasing traction, especially with new methodologies and protocols to mimic the 3D properties of environment. Recent studies have demonstrated that different geometrical states of the cell, such as its shape and spatial constraints, lead to significantly different transcriptional cellular responses, even when the cells are stimulated by the same biochemical factors (Mitra et al., 2017; Damodaran et al., 2018). In particular, recent efforts have focused in producing 3D *in vitro* environments that capture factors that normally are neglected or overlooked in 2D studies. Some studies use co-culturing in order to reproduce the interplay between different cell types, while simultaneously tuning the 3D culture setup to resemble the desired cues of the tissue microenvironment; an example is using spheroids of collagen matrix to mimic the interplay between fibroblast and cancer cells (Venkatachalapathy et al., 2020). Interestingly, fibroblasts were shown to sense the biochemical stimuli released by cancer cells, indicating a complex interplay between both cell types that affects the capability of fibroblasts to remodel the ECM and the capability of cancer cells to invade the surrounding tissue (Kalluri, 2016; Erdogan et al., 2017; Richards et al., 2017). A more physiological 3D environment can also be reproduced using gels, such as collagen I hydrogels to model pulmonary fibrotic tissues coupled to a fibrosis-on-chip model (Sundarakrishnan et al., 2018, 2019). It is also noteworthy that such 3D *in-vitro* microenvironments are also generally amenable to long-term culturing, enabling dynamic alteration of their properties to mimic the properties of tissue pathologies to study the fibroblast activation, which is still missing in 2D approaches. Thus, the development of tunable 3D physiological environment open new avenues for more in-depth studies into the coordination between different stimuli toward a better understanding and prevention of pathological progression.

## From Understanding to Controlling FMT

In conclusion, examining the role of mechanical and physical stimuli on cell behavior and fate is critical for understanding the pathophysiological state of the tissue. Specifically, the normal functioning of fibroblast throughout the organism can sensitively determine the difference between tissue healing or regeneration and progression to diseases, such as fibrotic diseases. Injuries, which result in mechanical and physical stresses to the tissue, can induce fibroblasts to switch their phenotype to a myofibroblastic state, characterized by elongated shape with the production of stress fibers. This phenotype transition cause reduced proliferation and migration, with an increased contractility (Hinz and Gabbiani, 2003; Hinz et al., 2004; Ward et al., 2008). As such, these myofibroblasts, which are normally is switched off in a more quiescent state, are responsible for the tissue stiffening as response to injuries such as in cardiac, lung, and liver diseases.

Physical and mechanical stimuli in the cellular microenvironment, such as topography, ligands spacing, and stiffness, have been identified as passive stimuli that allow the cells to complete the FMT by affecting the formation of FAs through the mechanotransductive pathway. This, in turn, causes cytoskeleton rearrangements, leading to α-SMA production, one of the hallmarks of myofibroblast (van Putten et al., 2016). Moreover, there are also active stimuli such as mechanical stretching that push the myofibroblasts to maintain their activated state, even when the biochemical part of the network causing the phenotype transition is switched off (Walker et al., 2020). This evidence unambiguously demonstrate that the environment where the cells reside has a very active role in regulating internal cell processes that induce different cell fate; mismatches lead to pathological states.

To study these processes systematically, *in-vitro* investigations involving production of substrates that allow researchers to control various combination of mechanical and physical cues has proven to be invaluable. At the same time, these efforts also highlight the possibility of using cellular environmental properties not only to gain in-depth understanding of the FMT process, but also to control and manipulate it. In particular, the formation and maturation of the FA complexes as well as the cytoskeletal rearrangements can be sensitively tuned using environmental cues and thus can present a unique toolset for tweaking the FMT process. We expect that the current rapid advances in the technologies to produce substrates with unprecedented control of the topographical and mechanical characteristics will further fuel the emergence of new methods and therapies to control cytoskeletal rearrangements, potentially allowing to reverse phenotype transition in fibrotic tissue and the progression of the disease.

## Author Contributions

MD'U and NK conceived, outlined, wrote, and approved the review. All authors contributed to the article and approved the submitted version.

## Conflict of Interest

The authors declare that the research was conducted in the absence of any commercial or financial relationships that could be construed as a potential conflict of interest.
